# Thickness Dependence on Interfacial and Electrical Properties in Atomic Layer Deposited AlN on *c*-plane GaN

**DOI:** 10.1186/s11671-018-2645-8

**Published:** 2018-08-10

**Authors:** Hogyoung Kim, Hee Ju Yoon, Byung Joon Choi

**Affiliations:** 1 Department of Visual Optics, Seoul National University of Science and Technology (Seoultech), Seoul, 01811 South Korea; 2 Department of Materials Science and Engineering, Seoul National University of Science and Technology (Seoultech), Seoul, 01811 South Korea

**Keywords:** Atomic layer deposited AlN, Interface state density, Reverse leakage current

## Abstract

The interfacial and electrical properties of atomic layer deposited AlN on n-GaN with different AlN thicknesses were investigated. According to capacitance–voltage (*C*–*V*) characteristics, the sample with a 7.4-nm-thick AlN showed the highest interface and oxide trap densities. When the AlN thickness was 0.7 nm, X-ray photoelectron spectroscopy (XPS) spectra showed the dominant peak associated with Al–O bonds, along with no clear AlN peak. The amount of remained oxygen atoms near the GaN surface was found to decrease for the thicker AlN. However, many oxygen atoms were present across the AlN layer, provided the oxygen-related defects, which eventually increased the interface state density. The barrier inhomogeneity with thermionic emission (TE) model was appropriate to explain the forward bias current for the sample with a 7.4-nm-thick AlN, which was not proper for the sample with a 0.7-nm-thick AlN. The reverse leakage currents for both the samples with 0.7- and 7.4-nm-thick AlN were explained better using Fowler–Nordheim (FN) rather than Poole–Frenkel emissions.

## Background

Because of large bandgap, high electron saturation velocity, and high breakdown field, III-nitride materials are of great interest not only for optoelectronic devices such as blue light emitting diodes (LEDs), laser diodes (LDs), and UV detectors but also for electronic devices such as high electron mobility transistors (HEMTs) and power devices [1–4]. Realizing high-performance GaN-based devices requires metal/GaN interface with a minimum interface state density, which can act as electron traps or limit to modulate the barrier heights according to metal work function by pinning the Fermi level [5, 6]. For other GaN-based device improvement techniques, some methods such as coalescence overgrowth of GaN nanocolumns, nonpolar *m*-plane GaN, nanoimprint GaN template, and semi-polar face GaN nanorods have also been demonstrated [7–11]. Among III-nitride compound semiconductors, aluminum nitride (AlN) can be applied to UV detectors, short-wavelength emitters and detectors, due to its high bandgap (∼ 6.2 eV), high thermal conductivity, high electric resistance, as well as low expansion at high temperatures [12, 13]. In addition, AlN can be deposited in a complementary metal-oxide-semiconductor (CMOS) compatible process by atomic layer deposition (ALD) (~ 300 °C), which is a big advantage. Polycrystalline- and amorphous ALD-grown AlN films can be used as dielectric layer for microelectronic devices [14]. Despite the progress of AlN growth techniques, ALD-grown AlN still reveals non-stoichiometric property which contains a large amount of oxygen-related impurities [15]. The amount of oxygen atoms in AlN can affect strongly the electrical and optical properties of AlN [16].

High-*k* dielectric oxides such as Al_2_O_3_ and HfO_2_ have been employed as a passivation layer in AlGaN/GaN high electron mobility transistors (HEMTs) [17, 18]. But the formation of Ga–O bonds at the Al_2_O_3_/(Al)GaN interface has been known to produce high density of deep (and slow) interface states [19]. As an alternative passivation material with low interface states, AlN has been considered for GaN-based devices due to its smaller lattice mismatch to GaN [20, 21]. In addition, modulation of electrical properties such as barrier heights in metal/semiconductor (MS) contacts by inserting very thin oxide layer has been reported in GaN [22, 23]. Increase of the barrier height in Pt/HfO_2_/GaN metal-insulator-semiconductor (MIS) diodes with a 5-nm-thick HfO_2_ layer was reported [22]. Insertion of a 3-nm MgO layer at a Fe/GaN interface was found to reduce the effective barrier height to 0.4 eV [23]. Still now, however, there is limited number of papers reporting on the engineered contact properties with ALD-grown AlN on GaN. In this work, we deposited AlN layers on n-GaN by ALD with different thicknesses and investigated the properties of AlN/n-GaN interface.

## Methods

### Materials and Device Fabrication

Hydride vapor phase epitaxy (HVPE)-grown, undoped, *c*-plane (0001) bulk GaN (thickness 300 μm, carrier concentration 5 × 10^14^ cm^−3^, threading dislocation density 1.5 × 10^7^ cm^−2^) purchased from Lumilog was used in this work. After cutting the wafer into small pieces, some of them were loaded into an ALD chamber after cleaning process in a HCl:H_2_O (1:1) solution. Then, the temperature was ramped up to 350 °C to deposit AlN layer. AlN thin films were deposited by thermal ALD system (manufacturer: CN-1 in Korea; model: Atomic Classic) using trimethylaluminum (TMA) and NH_3_ as precursors. Three different thick AlN layers (0.7, 1.5, and 7.4 nm) were prepared by varying the number of ALD cycles. The thicknesses of AlN film were measured using a FS-1 multi-wavelength ellipsometers (manufacturer: Film Sense in the USA; model: FS-1). To examine the electrical characteristics of the films, MIS diodes were fabricated with a Pt Schottky electrode (diameter 500 μm, thickness 50 nm) and an Al back contact (thickness 100 nm). As a reference, Pt/n-GaN Schottky diodes (i.e., without AlN layer) were also fabricated.

### Characterization

Temperature-dependent current–voltage (*I*–*V*–*T*) measurements were carried out with a HP 4155B semiconductor parameter analyzer after placing samples on a hot chuck connected with a temperature controller, and capacitance–voltage (*C*–*V*) measurements were performed using a HP 4284A LCR meter. X-ray photoelectron spectroscopy (XPS) measurements were carried out using a monochromatic Al *Κα* X-ray source to observe the formation mechanism at the AlN/GaN interface.

## Results and Discussion

**Fig. 1:**
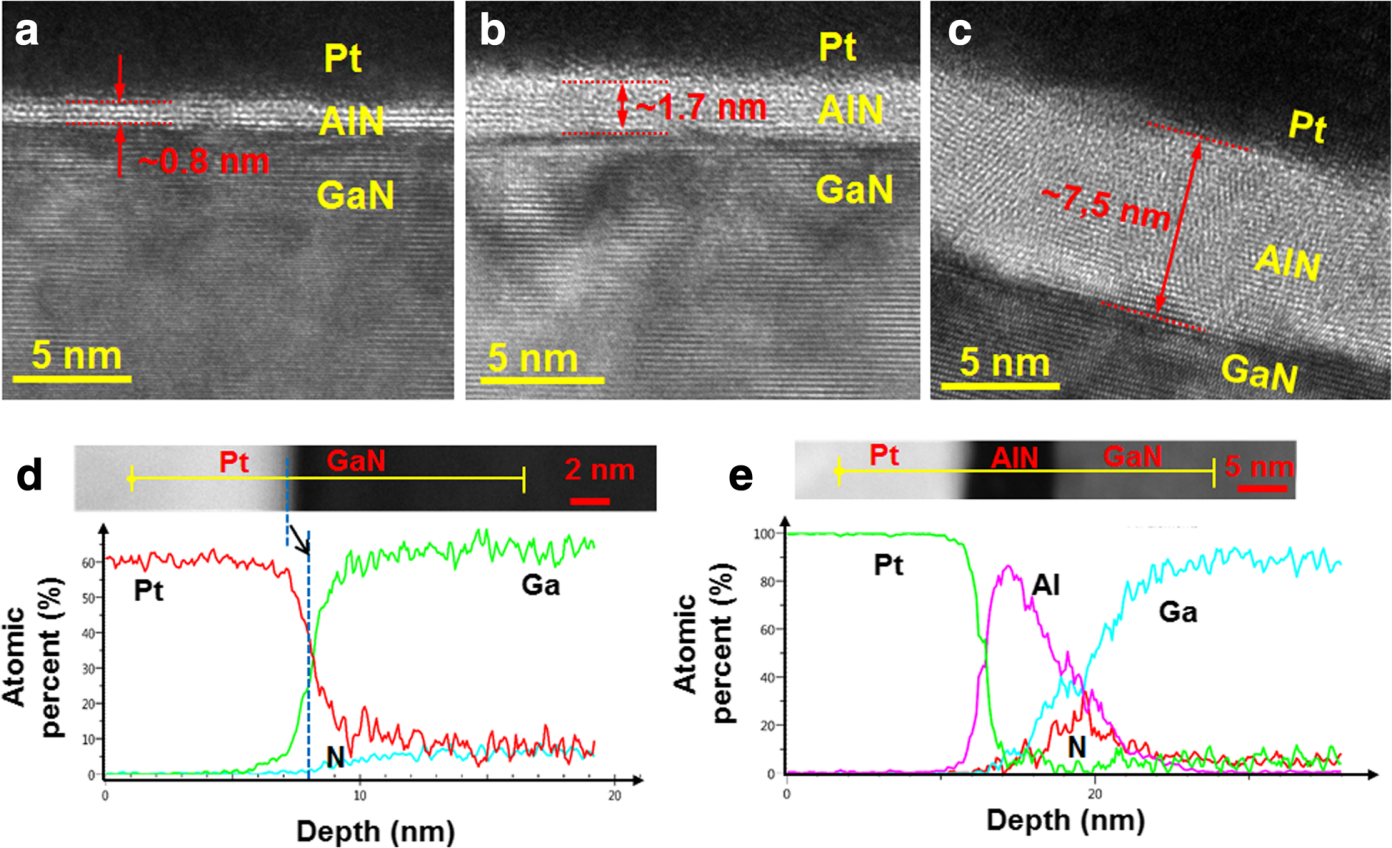
Cross-sectional scanning transmission electron microscopy (STEM) images with **a** 0.7-, **b** 1.5-, and **c** 7.4-nm-thick AlN. **d**, **e** Atomic percent vs. depth profiles obtained from energy dispersive X-ray spectroscopy (EDS) line scans for the samples with 0- and 7.4-nm-thick AlN, respectively

**Fig. 2:**
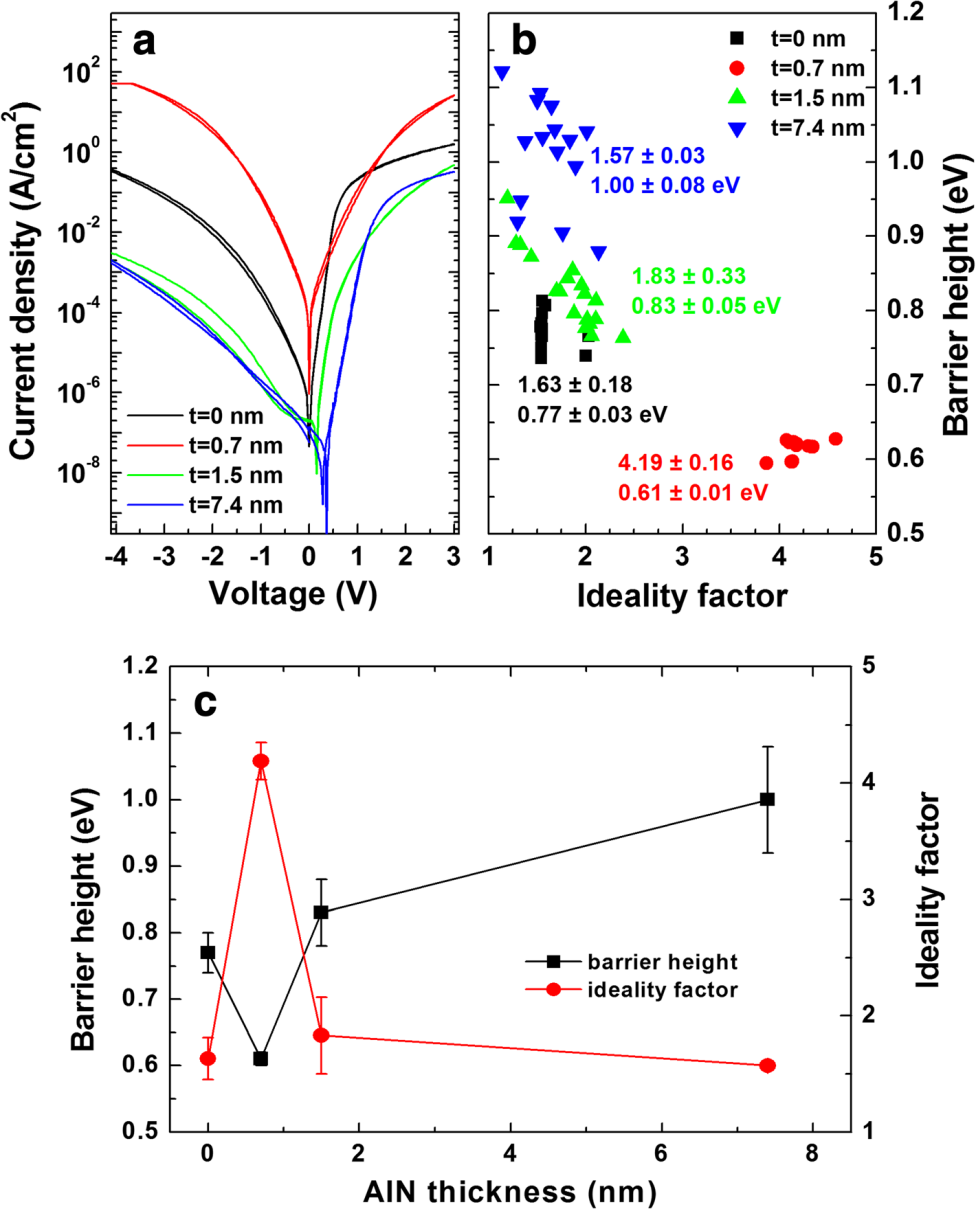
**a** Typical semilogarithmic current–voltage (*I*–*V*) characteristics. **b** Barrier height vs. ideality factor plots. **c** Barrier heights and ideality factors as a function of AlN thickness

Figure 1a–c shows the cross-sectional scanning transmission electron microscopy (STEM) images around the AlN layer. The estimated thicknesses of the AlN layers were similar to the values from ellipsometer. The typical semilogarithmic current density–voltage (*J*–*V*) curves are shown in Fig. 2a. Compared to the sample without AlN (i.e., reference sample), the current values increased for the sample with a 0.7-nm-thick AlN and decreased for the samples with 1.5- and 7.4 nm-thick AlN. Using the thermionic emission (TE) model [24], the forward bias current transport of a Schottky diode was analyzed to obtain both the barrier height and the ideality factor. The barrier heights were calculated to be 0.77 (± 0.03), 0.61 (± 0.01), 0.83 (± 0.05), and 1.00 (± 0.08) eV for the samples with 0-, 0.7-, 1.5-, and 7.4-nm-thick AlN, respectively. The ideality factors were found to be 1.63 (± 0.18), 4.19 (± 0.16), 1.83 (± 0.33), and 1.57 (± 0.03) for the samples with 0-, 0.7-, 1.5-, and 7.4-nm-thick AlN, respectively. With a 0.7-nm-thick AlN, the barrier height decreased and the ideality factor increased. With thicker AlN layers, the ideality factor was similar but the barrier height increased compared to the reference sample. It is seen in Fig. 2c that with increasing the AlN thickness the barrier height decreased first and then increased due to the tunneling resistance induced by thick AlN layer. This indicates that around 0.7 nm is a turning point for barrier height in terms of AlN thickness.

**Fig. 3:**
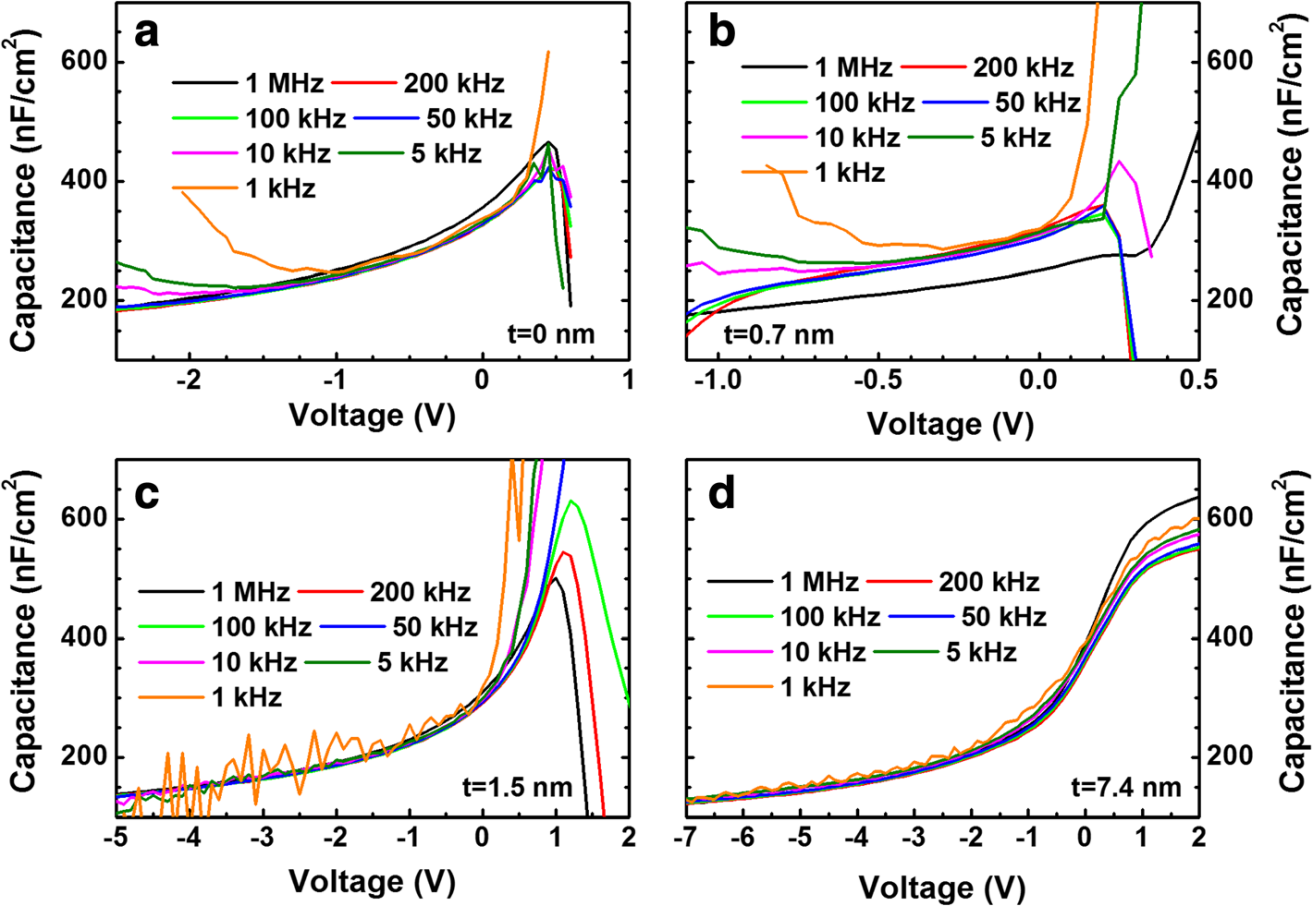
Capacitance–voltage (*C*–*V*) data measured at various frequencies for the samples with **a** 0-, **b** 0.7-, **c** 1.5-, and **d** 7.4-nm-thick AlN

Figure 3 shows the *C*–*V* curves measured at various frequencies. When the AlN thicknesses are 0 and 0.7 nm, the inversion in the capacitance values were observed below 10 kHz. Instead of inversion, deep depletion is normally observed for wide bandgap materials like GaN due to the low minority carrier (holes) generation rate [25, 26]. As shown in Fig. 3c, d, such inversion was not observed for thicker AlN layers. Here, it should be noted that in Au/GaN junction, no inversion was observed at low frequencies. Using deep level transient spectroscopy (DLTS), Auret et al. observed e-beam-induced defects in Pt/n-GaN Schottky junctions [27]. Here, we performed energy dispersive X-ray spectroscopy (EDS) measurements and the depth profiles for the samples with 0- and 7.4-nm-thick AlN are shown in Fig. 1d, e, respectively. It is clearly seen in Fig. 1d that Pt atoms diffused into the GaN layer, whereas the diffusion of Pt atoms into the GaN layer was suppressed effectively because of the AlN layer. Hence, it would be possible to suggest that Pt deposition-induced defects near the GaN surface produced the inversion capacitance at low frequencies and the formation of these defects were suppressed with a relatively thick AlN layer (> 1. 5 nm).

Except the sample with a 7.4-thick-AlN, all other samples showed the anomalous peak in the *C*–*V* curve with increasing the bias voltage, which were associated with the distribution of deep traps in the gap, the series resistance, and interface states [28, 29]. The frequency dispersion in the accumulation region is associated with the formation of an inhomogeneous layer at the interface. The capacitance of such layer acts in series with the oxide capacitance causing the dispersion in the accumulation [30]. The dispersion in depletion is due to the presence of interface states responding to applied frequency. If the time constants of the interface states are comparable to the frequency of small signal, the interface states make a contribution to the total capacitance such that the threshold capacitance increases with decreasing the frequency [31].

**Fig. 4:**
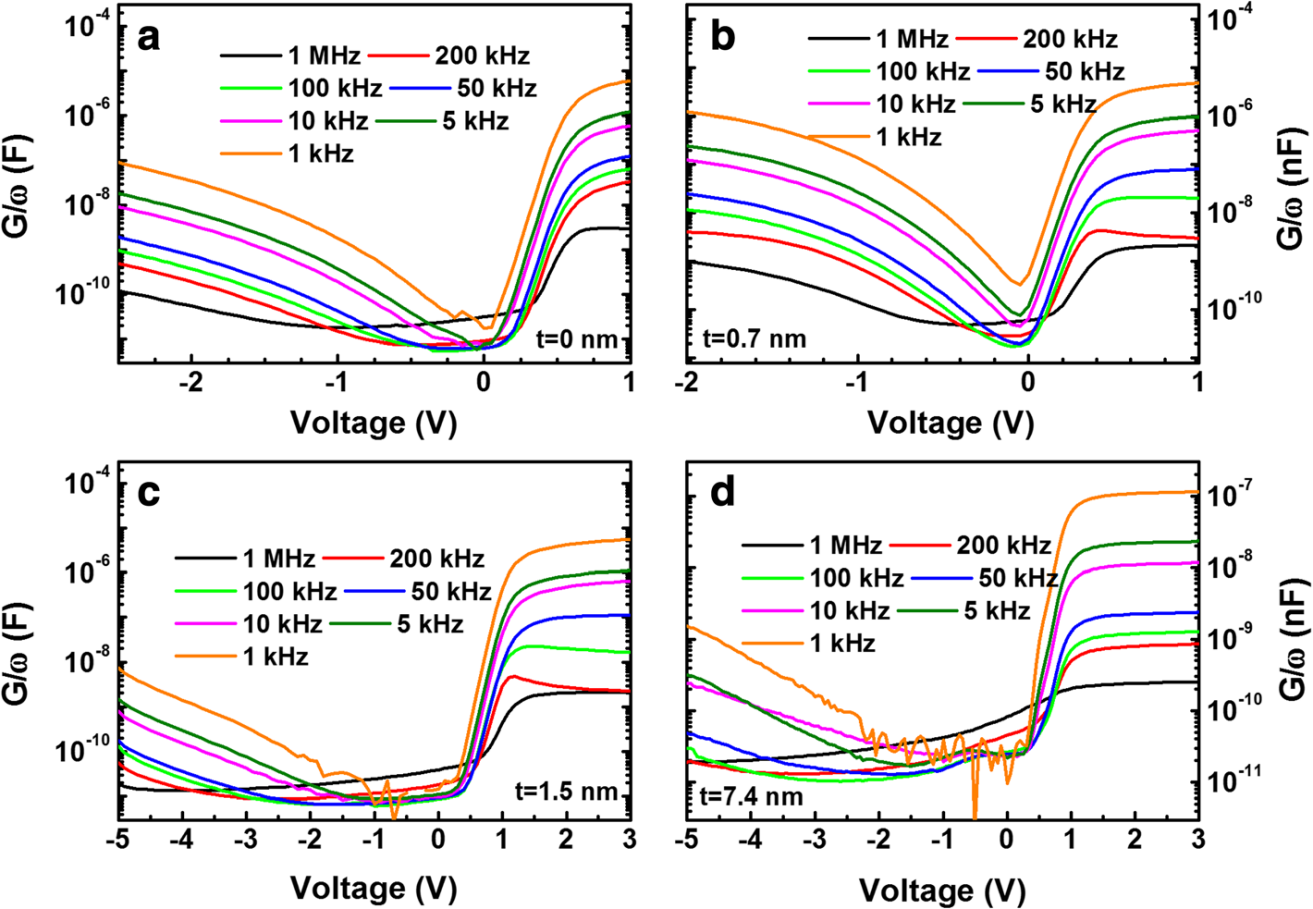
Conductance–voltage (*G*/*ω*–*V*) data measured at various frequencies for the samples with **a** 0-, **b** 0.7-, **c** 1.5-, and **d** 7.4-nm-thick AlN

Figure 4 shows the conductance–voltage (*G*/*ω*–*V*) curves measured at various frequencies. Under sufficiently high forward and reverse biases, the activated defects could communicate with neighboring interface states more effectively at low frequencies and hence increased the conductance. Approximately in the range of − 1 and 0 V, all the samples showed the increase in the conductance with increasing the frequency. This behavior became more prominent for the sample with a 7.4-nm-thick AlN. The increase in conductance with increasing the frequency was associated with the recombination centers promoting recombination current in the depletion region and the interface states providing charging and discharging current or hopping conduction process occurring at high frequency [32]. The results, therefore, indicate that interface states with various time constants are present for all the samples, and the presence of such defects are most significant for the sample with a 7.4-nm-thick AlN.

**Fig. 5:**
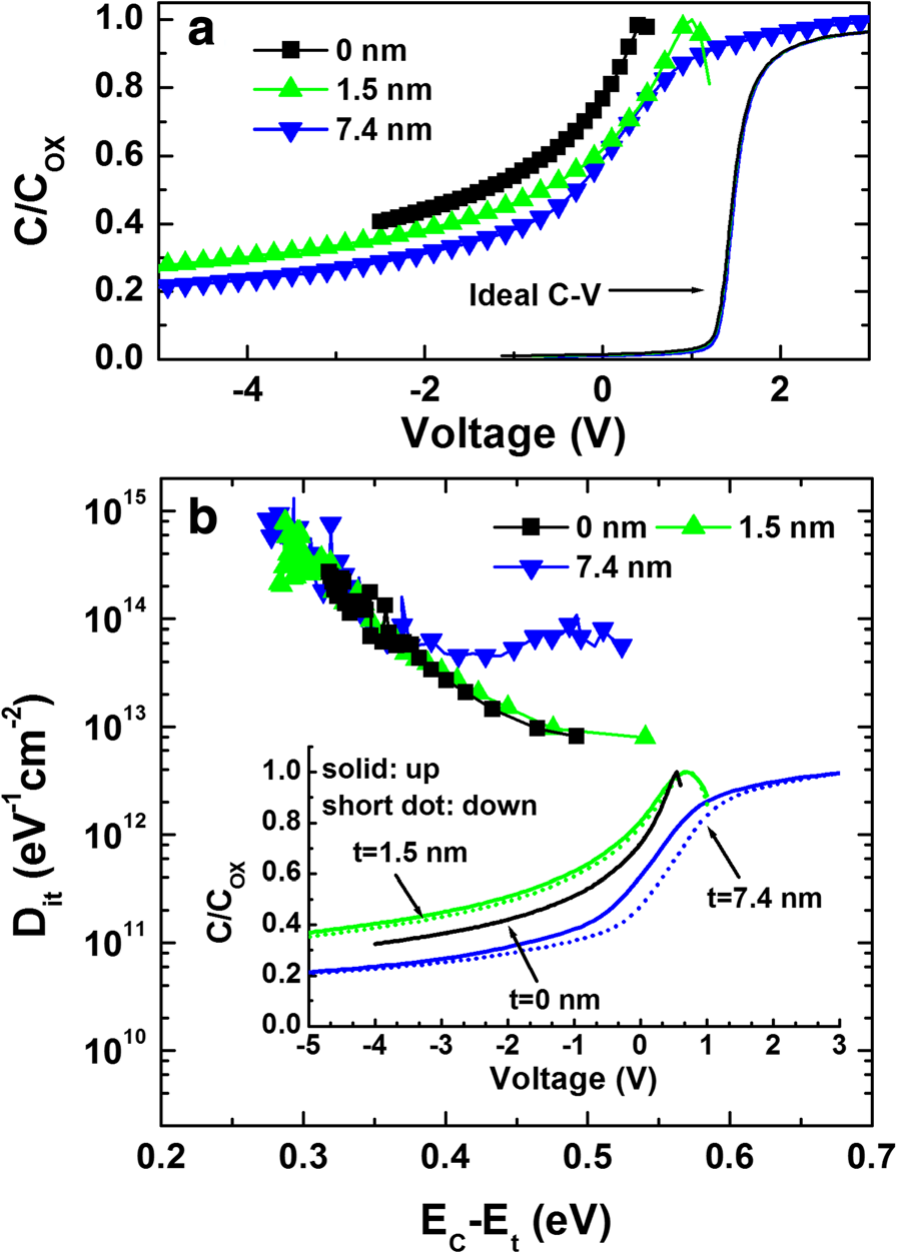
**a** Comparison of experimental capacitance–voltage (*C*–*V*) data measured at 1 MHz and ideal C–*V* data and **b** interface state density (*D*_it_) distributions determined by applying the Terman method for the samples with 0-, 1.5-, and 7.4-nm-thick AlN layer. The inset in **b** shows the *C*–*V* hysteresis plots measured at 1 MHz

As shown in Fig. 5a, an estimate of the interface state density (*D*_it_) was made by applying the Terman method to the experimental *C–V* curves measured at 1 MHz [33]. The obtained *D*_it_ vs. *E*_C_–*E*_t_ (location of the interface state) is presented in Fig. 5b. Here, we did not analyze the *C–V* curve from the sample with a 0.7-nm-thick AlN because the sample was leaky and the exact oxide capacitance (*C*_OX_) was not defined well. The sample with a 7.4-nm-thick AlN showed the highest interface state density, especially for *E*_C_–*E*_t_ > 0.4 eV. In addition, the average interface and oxide trap density (*Q*_T_) along the GaN bandgap (*E*_g_) were calculated by analyzing *C*–*V* hysteresis plots, using the flatband voltage shift (Δ*V*_FB_) through the equation *Q*_T_ = (*C*_OX_Δ*V*_FB_)/*qE*_g_ [34]. The small flatband voltage shift and the small hysteresis window indicate a low trap density. The inset in Fig. 4b shows *C*–*V* hysteresis plots. The trapped charge densities were calculated to be 4.2 × 10^9^, 9.3 × 10^9^, and 3.6 × 10^11^ cm^−2^ eV^−1^ for the samples with 0-, 1.5- and 7.4-nm-thick AlN, respectively. The hysteresis can originate from the interface traps of AlN/GaN and the border (or bulk) traps in the AlN layer. Like the Terman method, 7.4-nm-thick AlN layer revealed the highest interface and oxide trap density. Therefore, it is possible to suggest in this sample that the border traps in the AlN layer as well as the interface traps contributed significantly to the shift in *C*–*V* plots.

**Fig. 6:**
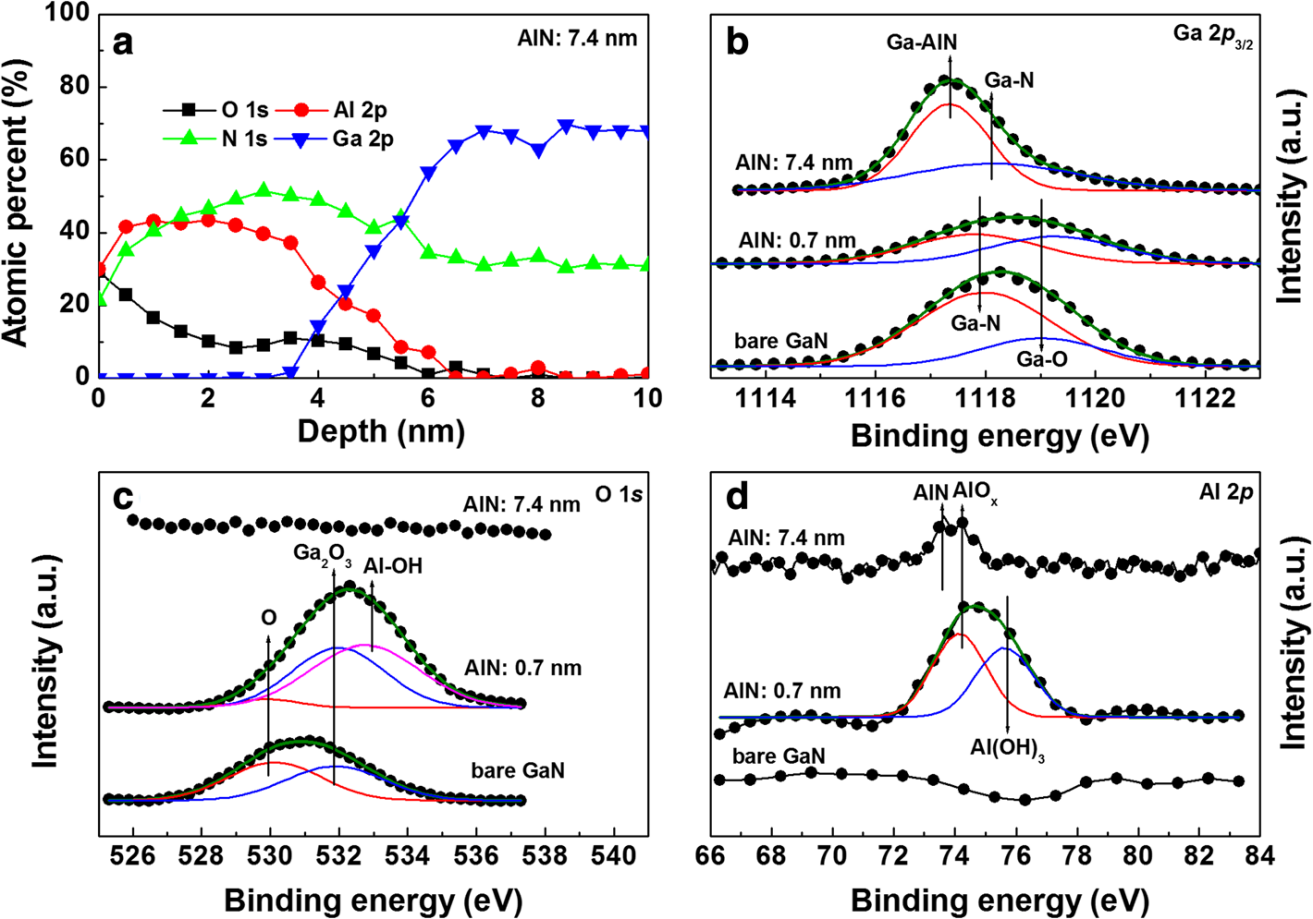
**a** XPS depth profiles for each element obtained from the sample with a 7.4-nm-thick AlN. XPS core-level spectra of **b** Ga 3*p*3/2, **c** O 1*s*, and **d** Al 2*p* for the samples with 0-, 1.5- and 7.4-nm-thick AlN

The chemical composition at the AlN/GaN interface was investigated using XPS measurements for two samples with 0.7- and 7.4-nm-thick AlN. Here, sputter etch treatment was performed on the sample with a 7.4-nm-thick AlN because the thickness of AlN was too thick to obtain the exact information near the AlN/InP interface. Bare GaN was also surface scanned as a reference. Figure 6a shows the XPS depth profiles for each element obtained from the sample with a 7.4-nm-thick AlN. The diffusion of Ga atoms into AlN layer was seen clearly. Fairly large amount of oxygen atoms were found to be present across the AlN layer. However, both O and Al atoms were not observed well near the AlN/GaN interface. The higher amount of oxygen near the AlN surface, compared to the AlN/GaN interface, indicates that a significant portion of it resulted from the atmospheric oxidation, not the ALD deposition process itself. We then selected the narrow scanned XPS spectra at one etch depth (thickness of the remained AlN was about 1.5–2.0 nm) and compared them to the data from other samples. Figure 6b shows the Ga 2*p*_3/2_ core-level spectra. The peaks at ~ 1118.0 eV and ~ 1119.2 eV for both the bare GaN and the sample with a 0.7-nm-thick AlN are associated with GaN and Ga_2_O_3_, respectively [35, 36]. The peak at ~ 1117.4 eV for the sample with a 7.4-nm-thick AlN is due to Ga bonded to AlN [37]. However, we cannot rule out the possibility that it may be from Ga_2_O peak (~ 1117.3 eV) [38].

As shown in Fig. 6c, the peaks at ~ 530.2 and ~ 531.9 eV are attributed to the chemisorbed O and Ga_2_O_3_, respectively [39]. In addition, the peak at ~ 532.8 eV is associated with Al–OH [40]. However, no peculiar peak was observed for the sample with a 7.4-nm-thick AlN at the selected depth. Similarly, no peak was observed at the deeper etch depths (not shown). When the AlN thickness is thin (0.7 nm), the chemisorbed oxygen atoms were removed but Al atoms bonded with OH. With increasing the AlN thickness, very little amount of oxygen atoms were present near the GaN surface region, indicating the cleaning up effect. However, large amount of oxygen atoms were present in the overgrown AlN region, provided oxide charges. O 1*s* core-level spectra at the etch depths where the amount of Ga atoms are negligible (about 0~3 nm from the AlN surface in Fig. 6a) were found to exhibit the dominant peak at ~ 531.8 eV, associated with Al_2_O_3_ [41]. This means that some portion of AlN layer is composed of Al_2_O_3_. As shown in Fig. 6d, the peak related with AlN is not observed well for the sample with a 0.7-nm-thick AlN. Rather, two peaks are observed at ~ 74.1 and ~ 75.6 eV, associated with AlO_*x*_ and Al–OH, respectively [42]. These Al–O bond-related peaks such as AlO_*x*_ and Al–OH can act as defects. The peak at ~ 73.6 eV for the sample with a 7.4-nm-thick AlN is associated with AlN [43].

**Fig. 7:**
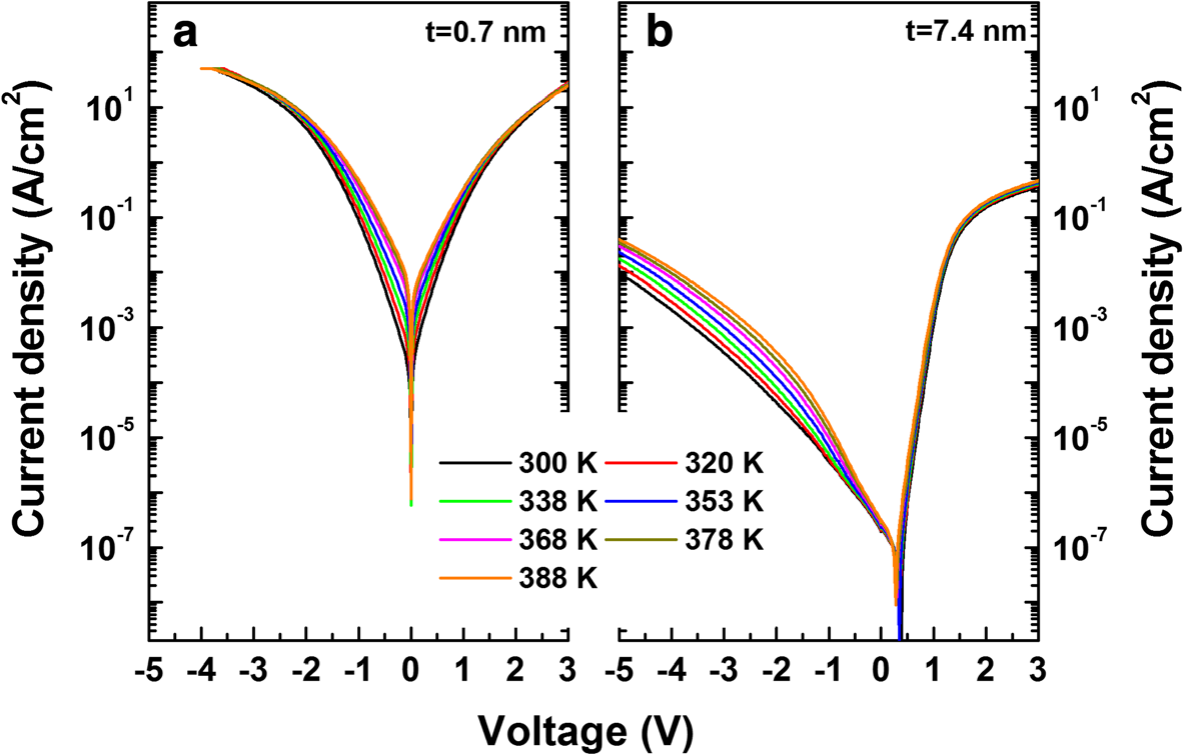
Temperature-dependent current density–voltage (*J*–*V*) characteristics for the samples with **a** 0.7- and **b** 7.4-nm-thick AlN

**Fig. 8:**
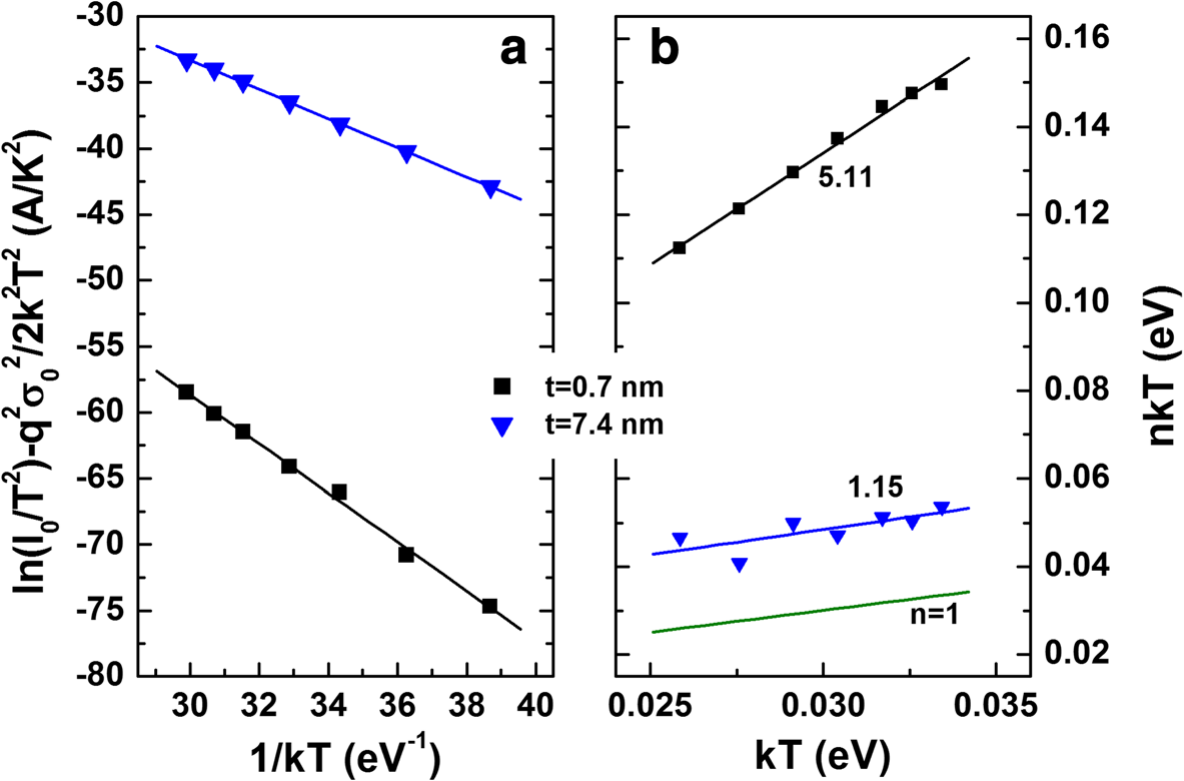
**a** Modified Richardson plots and **b** *nkT* vs. *kT* plots with the linear fits to the experimental data. In **b**, the line with a slope of 1 (*n* = 1) was also included as a reference

The current transport properties for the samples with 0.7- and 7.4-nm-thick AlN were investigated further using temperature-dependent current–voltage (*I*–*V*–*T*) measurements. As shown in Fig. 7, both the forward and the reverse bias current increased to a similar degree for the sample with a 0.7-nm thickness. For the sample with a 7.4-nm-thick AlN, however, the reverse leakage currents were more temperature dependent than the forward currents. Under reverse bias, higher temperature could cause hole thermal emission from the deep levels into the AlN valence band and, thus, introduced another supply of the electrons [44]. According to inhomogeneous barrier model [24], the temperature-dependent effective barrier height (*φ*_*B*_) is related with a zero-bias mean barrier height (${\overline{\varphi}}_B$) and a standard deviation (*σ*_0_) as ${\varphi}_B={\overline{\varphi}}_B-q{\sigma_0}^2/2 kT$. The *σ*_0_ values were obtained as 0.147 and 0.204 V for the samples with 0.7- and 7.4-nm-thick AlN, respectively. Using these values, the modified Richardson plots of ln(*I*_0_/*T*^2^) − *q*^2^*σ*_0_^2^/2*k*^2^*T*^2^ vs. 1/*kT* were obtained, shown in Fig. 8a. The intercepts at the ordinate produced the Richardson constants of A^**^ as 397.3 and 27.1 A cm^−2^ K^−2^ for the samples with 0.7- and 7.4-nm-thick AlN, respectively. The value for the sample with a 7.4-nm-thick AlN is similar to the theoretical value of 26.4 A cm^−2^ K^−2^ for n-GaN, indicating that the barrier inhomogeneity with the TE model can explain the current transport. However, for the sample with a 0.7-nm-thick AlN, the value is too high compared to the theoretical value, indicating that the TE model even including the barrier inhomogeneity cannot explain the current transport. Figure 8b shows the values of *nkT* as a function of *kT*. The straight line with slopes of 1.15 matches well to the experimental data for the sample with a 7.4-nm-thick AlN using TE model. For the sample with a 0.7-nm-thick AlN, however, the slope was found to be 5.11. Such large deviation from unity could arise from the interface states, insulator layer and tunneling current.

**Fig. 9:**
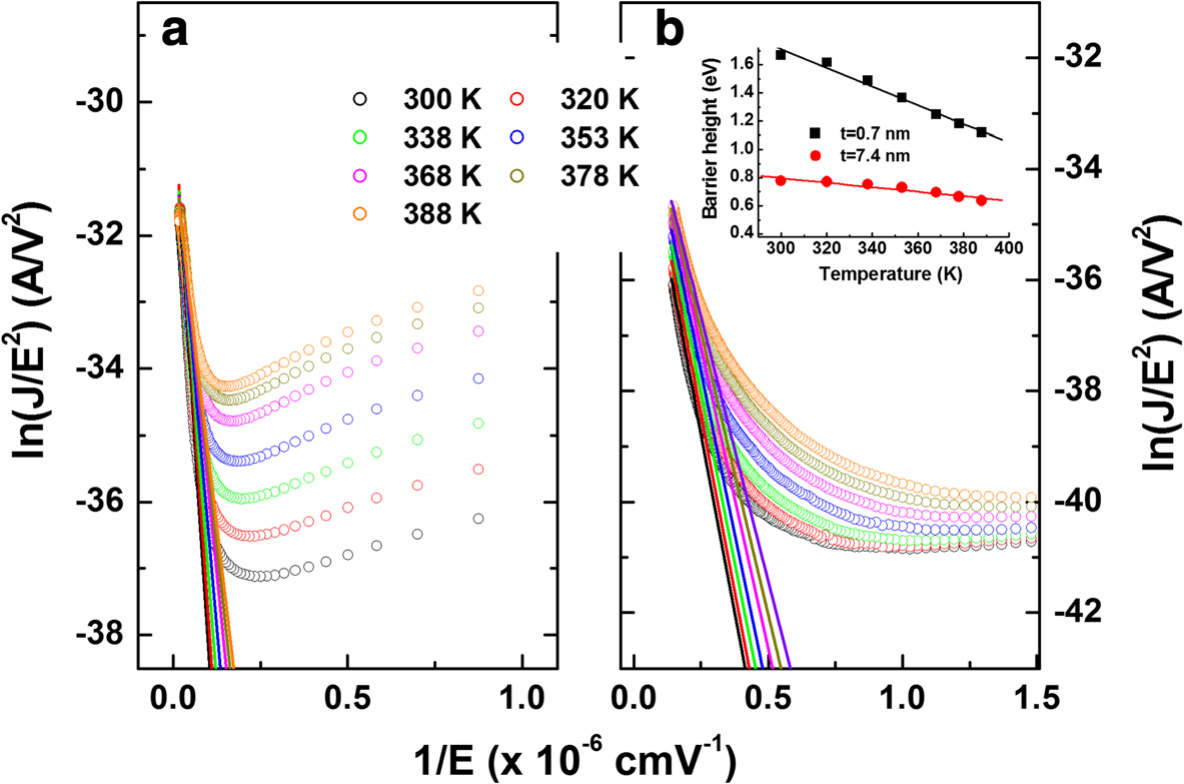
Fowler–Nordheim (FN) tunneling plots of ln(*J*/*E*^2^) vs. 1/*E* with the linear fits to the experimental data for the samples with **a** 0.7- and **b** 7.4-nm-thick AlN. The inset in **b** presents the calculated barrier heights as a function of temperature

The reverse leakage current density was analyzed using the Fowler–Nordheim (FN) tunneling model, given by [45].

1 $$ J=\alpha {E}^2\exp \left(-\beta /E\right) $$

where *α* = 1.54 × 10^−6^/*m*^∗^Φ_*B*_ and *β* = 6.83 × 10^−7^(*m*^∗^)^1/2^(Φ_*B*_)^3/2^; m^*^ (m^*^ = 0.30 for AlN [46]) is the effective electron mass in the insulator and Φ_*B*_is the tunneling barrier height. Figure 9a, b shows that FN emission was observed for the samples with 0.7- and 7.4-nm-thick AlN, when the bias voltages are higher than − 0.9 V and − 3 V, respectively. Higher voltage for the sample with a 7.4-nm-thick AlN is due to the fact that thicker AlN needs higher tunneling voltage. From the slope shown in Fig. 9, the tunneling barrier heights were determined for each temperature, which are presented in the inset in Fig. 9b. At room temperature, the barrier heights for the samples with 0.7- and 7.4-nm-thick AlN were calculated to be about 1.67 and 0.78 eV, respectively. These values are lower than the reported conduction band offset of 2.58 eV at AlN/GaN interface [47]. The poor interfacial quality near the AlN/GaN interface might produce such lower values. The lower barrier height for the sample with a 7.4-nm-thick AlN can also be associated with the high interface and oxide trap density near the AlN/GaN interface and oxygen-related defects in the AlN layer. As a result, trap-assisted tunneling occurred more easily and increased the reverse leakage current.

**Fig. 10:**
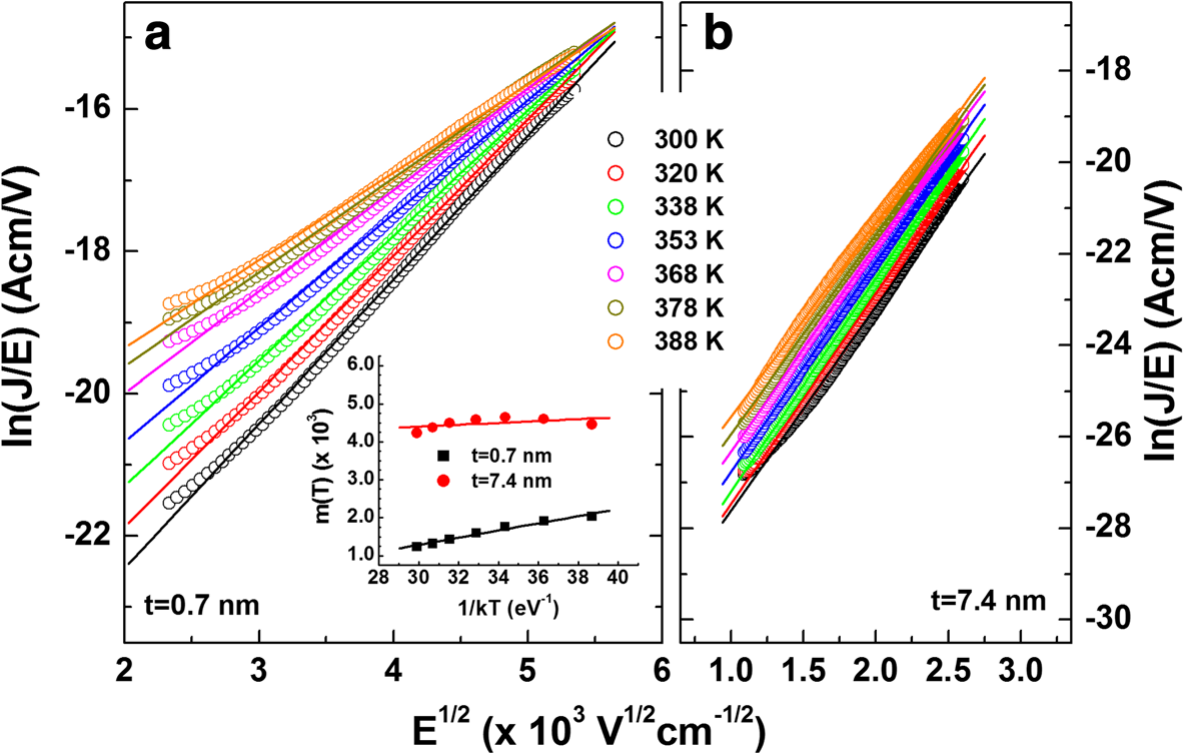
Poole–Frenkel (PF) emission plots of ln(*J*/*E*) vs. *E*^1/2/^ with the linear fit to the experimental data for the sample with **a** 0.7- and **b** 7.4-nm-thick AlN. The inset in **a** presents the calculated *m*(*T*) values vs. temperature

Poole–Frenkel (PF) emission model was also applied to the reverse leakage current, given by [48].

2 $$ \ln \left(J/E\right)=m(T){E}^{1/2}+b(T)\Big) $$

with

3 $$ m(T)=\frac{q}{kT}\sqrt{\frac{q}{{\pi \varepsilon}_0{\varepsilon}_{\mathrm{AlN}}}},b(T)=-\frac{q{\varphi}_t}{kT}+\ln C $$

where *ϕ*_t_ is the electron emission barrier height from the trap states, *ε*_AlN_ is the relative dielectric permittivity of the gate insulator at high frequency (*ε*_AlN_ 4.77 [49]), *ε*_0_ is the permittivity of free space, and *C* is a constant. The validity of the PF emission fitting was verified by checking the temperature dependence of the linear coefficient *m*(*T*) obtained from the lineal fit of the PF plots ln(*J*/*E*) as a function of *E*^1/2^ [50], which is shown in Fig. 10. From the *m*(*T*) values obtained from the linear fitting to these plots (inset in Fig. 10a), *ε*_AlN_ was found to be 64.9 and 959.0 for the samples with 0.7- and 7.4-nm-thick AlN, respectively. The obtained values are too high compared to the theoretical value of 4.77, which points out that PF emission cannot explain the current transport correctly for both samples. Hence, FN tunneling is more appropriate transport mechanism in the reverse leakage current.

Even though Eq. (1) contains no temperature dependence, the obtained barrier heights decreased with increasing the temperature. The slopes were obtained as − 6.67 meV/K and − 1.62 meV/K for the samples with 0.7- and 7.4-nm-thick AlN, respectively. It has been reported in SiO2/4H-SiC structure that the FN tunneling possesses a temperature dependence with a slope of − 7.6 meV/K [51]. The ejected electrons from the Pt electrode followed the Fermi–Dirac distribution [52], and thus, the reverse leakage current by the tunneling could also increase with temperature. In this case, the increase with temperature would be larger for thinner AlN layer.

Meanwhile, it has been reported that current transport mechanisms at high electric field cannot be explained solely by the FN tunneling [53, 54]. Even including the changes in the charges in the oxide and Fermi level of the substrate, and the electron energy distribution at the SiO_2_/SiC interface with temperature, the reverse leakage current in SiO_2_/4H-SiC was not explained satisfactorily [53]. It was proposed that thermally activated PF emission of trapped electrons from the interfacial electron traps contributes significantly to the increase in leakage current [54]. Therefore, reducing such defects in AlN during the ALD process is crucial in the AlN/GaN-based device performance, especially during the high-temperature operation.

As seen from the plot of barrier height vs. AlN thickness in Fig. 2c, Li et al. observed similar behavior in metal/n-Ge contacts with Y_2_O_3_ layers [55]. They attributed the reduction in the barrier height to the suppression of the unstable GeO_*x*_ growth and the passivation of dangling bonds on the Ge surface. Karpov et al. inserted Si_3_N_4_ layer into Ni/n-GaN contacts and found that the barrier height decreased from 0.78 to 0.27–0.30 eV with a Si_3_N_4_ layer. The results were explained by the dipole formation at the Si_3_N_4_/GaN interface [56]. Further, Zheng et al. investigated the contact resistance vs. Al_2_O_3_ thickness in Al/n-SiC structure and found that the interface dipole started to form at the thickness of 1.98 nm [57]. Above this thickness, the contact resistance decreased first due to the dipole effect and then increased due to the increased tunneling resistance. According to XPS data in Fig. 6, the formation of AlN layer is unclear for the sample with a 0.7-nm-thick AlN. Hence, the reduction of barrier height with a 0.7-nm-thick AlN is more likely due to the passivation effect rather than the formation of interface dipole.

Dry etching process such as inductively couple plasma (ICP) etching is widely used in GaN-based devices due to the chemical stability of GaN [58], even though ultraviolet-enhanced wet chemical etching was demonstrated [59]. However, dry etching process can induce damage on the GaN surface, increasing the leakage current and degrading the rectifying behavior. Post etch treatment using thermal annealing and KOH solution after reactive ion etching (RIE) was found to effectively remove the surface damage on GaN [60]. Considering the results so far, we suggest that AlN deposition (larger than 1 nm) can be applied to reduce the damage on the etched GaN surface, which is expected to increase the interface quality and the rectifying characteristics further.

## Conclusions

We have investigated the interfacial and electrical properties of atomic layer deposited AlN on n-GaN with different AlN thicknesses. According to capacitance–voltage (*C*–*V*) characteristics, the sample with a 7.4-nm-thick AlN showed the highest interface and oxide trap density. According to X-ray photoelectron spectroscopy (XPS) measurements, the sample with a 0.7-nm-thick AlN revealed a dominant peak related with Al–O bonds, with no clear peak associated with AlN. The remained oxygen atoms near the GaN surface were found to be very little for the sample with a 7.4-nm-thick AlN. On the other hand, many oxygen atoms were found to be present across the AlN layer, which provided the oxygen-related defects in the AlN layer. Analyses on the reverse leakage current revealed that Fowler–Nordheim (FN) rather than Poole–Frenkel (PF) emission were more appropriate to explain the current transport for the samples with 0.7- and 7.4-nm-thick AlN.

## Abbreviations

ALD: Atomic layer deposition
AlN: Aluminum nitride
*C*–*V*: Capacitance–voltage
FN: Fowler–Nordheim
*J*–*V*: Current density–voltage
PF: Poole–Frenkel
TE: Thermionic emission
XPS: X-ray photoelectron spectroscopy
